# Synthetic double inversion recovery imaging for rectal cancer T staging evaluation: imaging quality and added value to T2-weighted imaging

**DOI:** 10.1186/s13244-024-01796-4

**Published:** 2024-10-24

**Authors:** Zi Wang, Zhuozhi Dai, Xinyi Zhou, Jiankun Dai, Yuxi Ge, Shudong Hu

**Affiliations:** 1 Department of Radiology, Affiliated Hospital of Jiangnan University, Wuxi, Jiangsu China; 2 Department of Radiology, Shantou Central Hospital, Shantou, Guangdong China; 3 Department of Pathology, Affiliated Hospital of Jiangnan University, Wuxi, Jiangsu China; 4 GE Healthcare, MR Research China, Beijing, China; 5 Institute of Translational Medicine, Jiangnan University, Wuxi, Jiangsu China

**Keywords:** Rectal neoplasms, Neoplasm staging, Magnetic resonance imaging, Synthetic imaging

## Abstract

**Objective:**

To assess the image quality of synthetic double inversion recovery (SyDIR) imaging and enhance the value of T2-weighted imaging (T2WI) in evaluating T stage for rectal cancer patients.

**Methods:**

A total of 112 pathologically confirmed rectal cancer patients were retrospectively selected after undergoing MRI, including synthetic MRI. The image quality of T2WI and SyDIR imaging was compared based on signal-to-noise ratio (SNR), contrast-to-noise ratio (CNR), overall picture quality, presence of motion artifacts, lesion edge sharpness, and conspicuity. The concordance between MRI and pathological staging results, using T2WI alone and the combination of T2WI and SyDIR for junior and senior radiologists, was assessed using the Kappa test. The area under the receiver operating characteristic curve (AUC) was used to assess the diagnostic efficacy of extramural infiltration in rectal cancer patients.

**Results:**

No significant differences in imaging quality were observed between conventional T2WI and SyDIR (*p* = 0.07–0.53). The combination of T2WI and SyDIR notably improved the staging concordance between MRI and pathology for both junior (kappa value from 0.547 to 0.780) and senior radiologists (kappa value from 0.738 to 0.834). In addition, the integration of T2WI and SyDIR increased the AUC for diagnosing extramural infiltration for both junior (from 0.842 to 0.918) and senior radiologists (from 0.917 to 0.938).

**Conclusion:**

The combination of T2WI and SyDIR increased the consistency of T staging between MRI and pathology, as well as the diagnostic performance of extramural infiltration, which would benefit treatment selection.

**Critical relevance statement:**

SyDIR sequence provides additional diagnostic value for T2WI in the T staging of rectal cancer, improving the agreement of T staging between MRI and pathology, as well as the diagnostic performance of extramural infiltration.

**Key Points:**

- Synthetic double inversion recovery (SyDIR) and T2WI have comparable image quality.
- SyDIR provides rectal cancer anatomical features for extramural infiltration detections.
- The combination of T2WI and SyDIR improves the accuracy of T staging in rectal cancer.

**Graphical Abstract:**

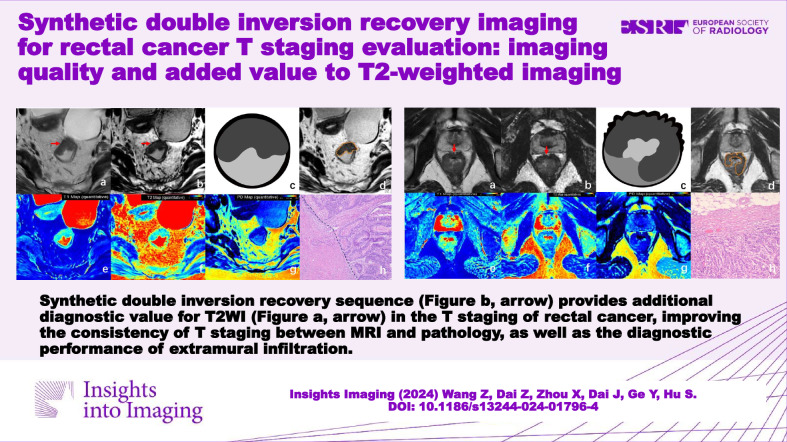

## Introduction

Rectal cancer, a globally prevalent gastrointestinal malignancy, poses an escalating menace to human well-being [1]. Total mesorectal excision has emerged as the quintessential therapeutic approach. According to the American National Comprehensive Cancer Network and Chinese guidelines for colorectal cancer, preoperative neoadjuvant chemoradiotherapy is the gold standard for patients afflicted with T3-4 or node-positive tumors, as it effectively down-stages the disease, mitigates local recurrence rates, and facilitates sphincter-preserving interventions [2, 3]. Underestimating the stage of rectal cancer may result in excluding preoperative chemoradiotherapy, thereby heightening the likelihood of local recurrence. Conversely, overestimating the stage may prompt unwarranted administration of preoperative chemoradiotherapy, potentially causing significant functional repercussions [2, 3]. Consequently, precise T staging is paramount for the judicious selection of treatment modalities.

T2-weighted imaging (T2WI) is the most crucial magnetic resonance imaging (MRI) sequence for evaluating T staging and anatomical structures in rectal cancer. Previous studies demonstrated that its diagnostic accuracy ranges from 62–85% [4, 5], with its precision is dependent on the radiologist’s expertise [6]. The limitations of T2WI in diagnosing rectal cancer T staging encompass its inability to differentiate between fibrosis and extramural infiltration, impeding the distinction between early-stage T3 tumors and T2 tumors [7]. Consequently, substantial experience and high-quality images are requisite for discerning the nuanced findings that facilitate T staging differentiation.

Synthetic MRI (SyMRI) represents an innovative technology capable of synthesizing multiple contrast-weighted images in a single scan, encompassing T1WI, T2WI, proton density-weighted imaging, double inversion recovery (DIR) images, and more [8]. DIR images offer enhanced tissue contrast by simultaneously nullifying signals from two distinct tissues when two 180° inversion pulses precede a conventional spin-echo acquisition, which has been applied to prostate cancer [9], synovitis of the knee [10], and gray-white matter contrast in the brain [11]. The DIR-MRI sequence attenuates cerebrospinal fluid and white matter in the brain, achieving a superior definition between gray and white matter and being effective in the diagnosis of multiple sclerosis and cerebral gray matter ectopia [12, 13]. Compared to conventional DIR, synthetic DIR (SyDIR) allows for manual adjustment of inversion time (TI) to optimize the visualization of specific tissues. For example, in the knee joint, SyDIR has the potential to accentuate the synovium without using contrast agents, by simultaneously suppressing water and fat [14]. Therefore, we speculate that SyDIR can be used to enhance tissue contrast between rectal tumors and surrounding fat in order to clearly show the depth of tumor invasion.

The objective of this study was to comprehensively evaluate the imaging quality and clinical significance of SyDIR in the assessment of T-stage and extramural infiltration of rectal cancer.

## Materials and methods

### Patients

**Fig. 1:**
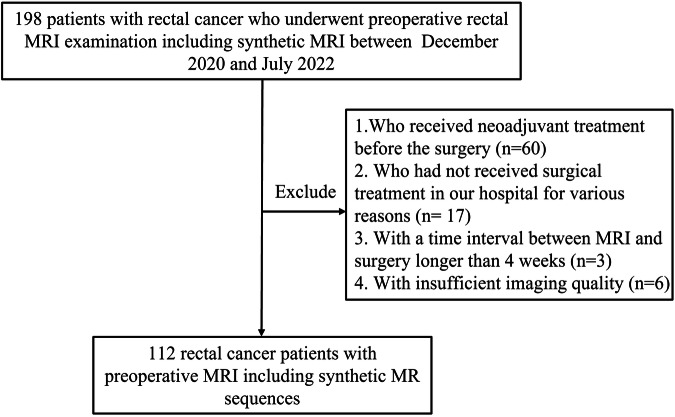
Flowchart of the patient selection process

The Institutional Review Board approved this retrospective, single-center study, and this study comprises an ad-hoc analysis of data from a previous prospective study with informed consent. A total of 198 patients with pathologically confirmed rectal adenocarcinoma, defined as located within 15 cm from the anal verge, underwent rectal MRI examinations using SyMRI sequences between December 2020 and July 2022, were enrolled. The exclusion criteria were as follows: (1) patients who received neoadjuvant treatment before surgery; (2) patients who did not undergo surgical treatment at our hospital for various reasons; (3) instances where the time interval between the MRI examination and surgery exceeded 4 weeks; (4) cases with insufficient imaging quality. Ultimately, 112 patients were incorporated into the study. The flowchart depicting the study cohort is illustrated in Fig. 1.

### MRI acquisition

MRI was performed using a 3.0-T scanner (SIGNA^TM^ Architect; GE Healthcare, USA) outfitted with a 16-channel phased array body coil. Participants got glycerin enemas to prepare their bowels for the test. 20 mg of anisodamine was administered intramuscularly 20 min before the examination (with the exception of patients with contraindications) to reduce intestinal motility. Conventional contrast-weighted images with high-resolution T2-weighted imaging (HR-T2WI) were obtained in the sagittal, axial, and coronal positions according to the following parameters: the field of view, 24 × 24 cm to 30 × 30 cm; flip angle, 111°; matrix size, 320 × 224; slice thickness/spacing, 3 mm/0.3 mm; sections, from 18 to 28; acquisition time, 2 min 24 s to 2 min 45 s. SyMRI was performed before the administration of intravenous contrast agents. For axial SyMRI, two echo times (19.5/97.3 ms) and four saturation delay times (210/610/1810/3810 ms) were used according to the following parameters: the viewing area, 24 × 24 cm; flip angle, 111°; matrix, 320 × 224; thick, 3 mm; spacing, 0.3 mm; sections, from 18 to 28; acquisition time, 4 min 20 s. Using SyMRI 8.0 software (Synthetic MR, Linkoping, Sweden), the synthetic images were processed. Quantitative pictures such as SyT2WI (Synthetic T2-weighted imaging) and SyDIR were also produced using this software. Referring to the application of DIR in the prostate as described in the above references (TI1 = 3400 ms, TI2 = 450 ms) [9], the SyDIR sequence was optimized using SyMRI 8.0 software. A longer TI1 of 3750 ms was chosen to suppress water interference in tumor display since the rectum is a mesorectal cavity organ with a high water content. By adjusting TI2 to 475 ms, clear rectal anatomy was achieved with a black line-like “bounce point” artifact at the fat-tumor interface [15]. Ultimately, TI values of 3750 ms (TI1) and 475 ms (TI2), along with a TR value of 15,000 ms, were deemed optimal for rectal assessment in this study.

### Image quality analysis

Two radiologists with 4 and 25 years of experience in rectal imaging, who were blinded to the study design, clinical patient data, diagnoses, and final pathology reports, assessed the image quality of T2WI and SyDIR, followed by a repeat evaluation by the junior radiologist after a two-week interval. The subjective image quality assessment includes the following four points using 5-Likert-type scales from 1 to 5: (1) overall image quality (1 = non-diagnostic, 2 = poor, 3 = moderate, 4 = good, and 5 = excellent). (2) lesion edge sharpness (1 = not sharp, 2 = merely sharp, 3 = intermediate sharp, 4 = good sharp, and 5 = excellent sharp) (3) presence of motion artifacts (1 = severe, difficult to diagnose, 2 = a little severe, 3 = moderate, 4 = mild, and 5 = absent) (4) lesion conspicuity (1 = not detectable, 2 = merely recognizable, 3 = moderate recognizable, 4 = good, and 5 = excellent). The schematic diagram of the image quality scoring is shown in the Supplementary Fig. 1. The order of patients was randomized, as was the order of review of T2WI or SyDIR.

The same two radiologists used a vendor-provided workstation (AW4.7; GE Healthcare) to manually draw region of interest (ROI) on axial T2WI and SyDIR in order to assess the objectivity of the image quality, with the junior radiologist reevaluating the images after two weeks. The ROI for the normal tissue was the muscle of the obturator internus. The 10 mm-diameter of the background ROI was positioned within the field of view but outside the body surface. The average and range of signal intensity were produced for each ROI. Signal-to-noise ratio (SNR) and contrast-to-noise ratio (CNR) are calculated using the following formulas [16]:

$${{{\rm{SNR}}}} 	= {{{\rm{S}}}}_{{{\rm{tumor}}}}/{{{\rm{SD}}}}_{{{\rm{background}}}},\\ {{{\rm{CNR}}}} 	 = {{{\rm{|S}}}}_{{{\rm{tumor}}}}-{{{\rm{S}}}}_{{{\rm{tissue}}}}|/{{{\rm{SD}}}}_{{{\rm{background}}}}$$

where SD_background_ stands for the standard deviation of the background noise, S_tissue_ refers for the mean signal intensity of the obturator internus muscle, and S_tumor_ is the mean signal intensity within the tumor.

### Imaging analysis and measurement

The same two radiologists (S.H. and Z.D.) independently assessed the T stage of rectal cancer while being unaware of the clinical and pathological data. The T stage was first evaluated using HR-T2WI, and two weeks later, the T stage was evaluated using HR-T2WI and SyDIR imaging. For SyMRI-derived parameters, ROI was manually delineated on the single slice with the largest tumor diameter on SyT2WI image, avoiding intestinal contents and necrosis. The corresponding T1, T2, and proton density (PD) values of the tumors were automatically calculated. The eighth edition of the American Joint Committee on Cancer TNM staging system was used to evaluate pathological (p) T-stage surgical specimens. Patients were divided into groups according to their pT characteristics: pT1-2 (non-extramural infiltration) and pT3-4 (extramural infiltration), which are likely to have an impact on how their treatments progress.

### Statistical analysis

The Kappa consistency test was used to assess MR and pathologic T-staging of rectal cancer, as well as subjective image quality scores between different observers, with the following interpretation: 0.21–0.40 (fair), 0.41–0.60 (moderate), 0.61–0.80 (good), and 0.81–1.00 (excellent). Intraclass correlation coefficient was employed to investigate the interobserver agreement of the SNR, CNR, and quantitative values (0.21–0.40, fair; 0.41–0.60, moderate; 0.61–0.80, good; 0.81–1.00, excellent). Depending on the normality of the data distribution, the independent-sample *t*-test or the Mann–Whitney *U* test was used to compare groups for continuous variables. Wilcoxon signed-rank test was used to compare the image quality scores, SNR, and CNR between T2WI and SyDIR. The effectiveness of the diagnostic process was assessed using the area under the receiver operating characteristic (ROC) curve (AUC); the AUCs variation was compared using DeLong’s test. Herein, statistical significance was defined as *p* < 0.05. The Statistical Package for the Social Sciences (version 22.0; IBM, Armonk, NY) and MedCalc Statistical Software (MedCalc 18.2, Belgium) were used for all statistical analyses.

## Results

### Clinical characteristics and MR features

All 112 patients (male 68, female 44) were included in the present study. The patients’ age ranged from 37 to 81 years (mean 65 years). Among all the cases, poorly differentiated rectal adenocarcinoma accounted for 24.1% (27/112) and mucinous adenocarcinoma accounted for 9.8% (11/112) of the cases. The mean length and thickness of the tumor were 3.96 ± 1.60 cm and 2.48 ± 1.44 cm. Detailed characteristics of the study population are listed in Table 1.

**Table 1:** Clinical characteristics

Characteristics	Number of patients (%)
Age (years), Mean ± SD	65 ± 9
Gender	
Male	68 (60.7)
Female	44 (39.3)
Surgical resection type	
Low anterior or anterior resection	65 (58.0)
Abdominoperineal resection	37 (33.0)
Hartman’s resection	10 (8.8)
Differentiation	
Well/Moderate	85 (75.9)
Poor	27 (24.1)
Pathological types	
Adenocarcinoma (non-specific)	101 (90.2)
Mucinous adenocarcinoma	11 (9.8)
MTL (Mean ± SD, cm)	3.96 ± 1.60
Thickness (cm)	2.48 ± 1.44
Tumor location (No. %)	
Upper	20 (17.9)
Middle	62 (55.4)
Lower	30 (26.8)
pT stage	
pT1-2	45 (40.2)
pT3	43 (38.4)
pT4	24 (21.4)
pN stage	
N (−)	68 (60.7)
N (+)	44 (39.3)
pEMVI	
Absent	79 (70.5)
Present	33 (29.5)

*MTL* maximum tumor length, *pEMVI* pathological extramural venous invasion

### Image quality and interobserver reliability

There were no statistically significant differences between conventional T2WI and SyDIR in SNR, CNR, overall image quality, absence of motion artifacts, sharpness of the lesion edge, and lesion conspicuity (*p* = 0.07–0.53 for all comparison pairs, Table 2). The interobserver agreements of SNR, CNR, overall image quality, absence of motion artifacts, sharpness of the lesion edge, and lesion conspicuity for SyDIR were moderate (kappa values = 0.64–0.79). The results of interobserver agreements are summarized in Table 2.

**Table 2:** Comparison of image quality between T2WI and synthetic DIR

	T2WI	Synthetic DIR	*Z/p* value	Intra-observer agreement of SyDIR (95% CI)	Interobserver agreement of SyDIR (95% CI)
SNR					
Reader 1	27.94 ± 4.29	27.39 ± 5.80	1.25, 0.21	0.87 (0.80–0.92)	0.73 (0.61–0.81)
Reader 2	28.21 ± 4.49	28.07 ± 5.24	1.81, 0.07		
CNR					
Reader 1	19.02 ± 3.14	19.43 ± 4.17	1.74, 0.08	0.89 (0.85–0.93)	0.79 (0.69–0.85)
Reader 2	19.57 ± 3.41	19.93 ± 4.12	1.53, 0.13		
Overall picture quality					
Reader 1	4.72 ± 0.51	4.71 ± 0.53	1.41, 0.16	0.91 (0.82–0.99)	0.74 (0.61–0.86)
Reader 2	4.66 ± 0.51	4.63 ± 0.52	1.73, 0.08		
Presence of motion artifacts					
Reader 1	4.68 ± 0.54	4.65 ± 0.58	1.73, 0.08	0.80 (0.68–0.93)	0.64 (0.49–0.79)
Reader 2	4.59 ± 0.56	4.61 ± 0.54	0.63, 0.53		
Conspicuity					
Reader 1	4.62 ± 0.62	4.61 ± 0.65	1.00, 0.32	0.86 (0.76–0.96)	0.66 (0.52–0.80)
Reader 2	4.55 ± 0.60	4.57 ± 0.58	0.63, 0.53		
Lesion edge sharpness					
Reader 1	4.63 ± 0.57	4.60 ± 0.59	1.63, 0.10	0.78 (0.65–0.91)	0.64 (0.50–0.78)
Reader 2	4.56 ± 0.58	4.60 ± 0.55	1.27, 0.21		

*SNR* signal-to-noise ratio, *CNR* contrast-to-noise ratio, *T2WI* T2-weighted imaging, *DIR* double inversion recovery, *SyDIR* synthetic double inversion recovery

### Evaluation of T staging

On the SyDIR, rectal cancer lesions showed intermediate high signal intensity, water and fat were partially suppressed with low and slightly high signals, respectively.

**Fig. 2:**
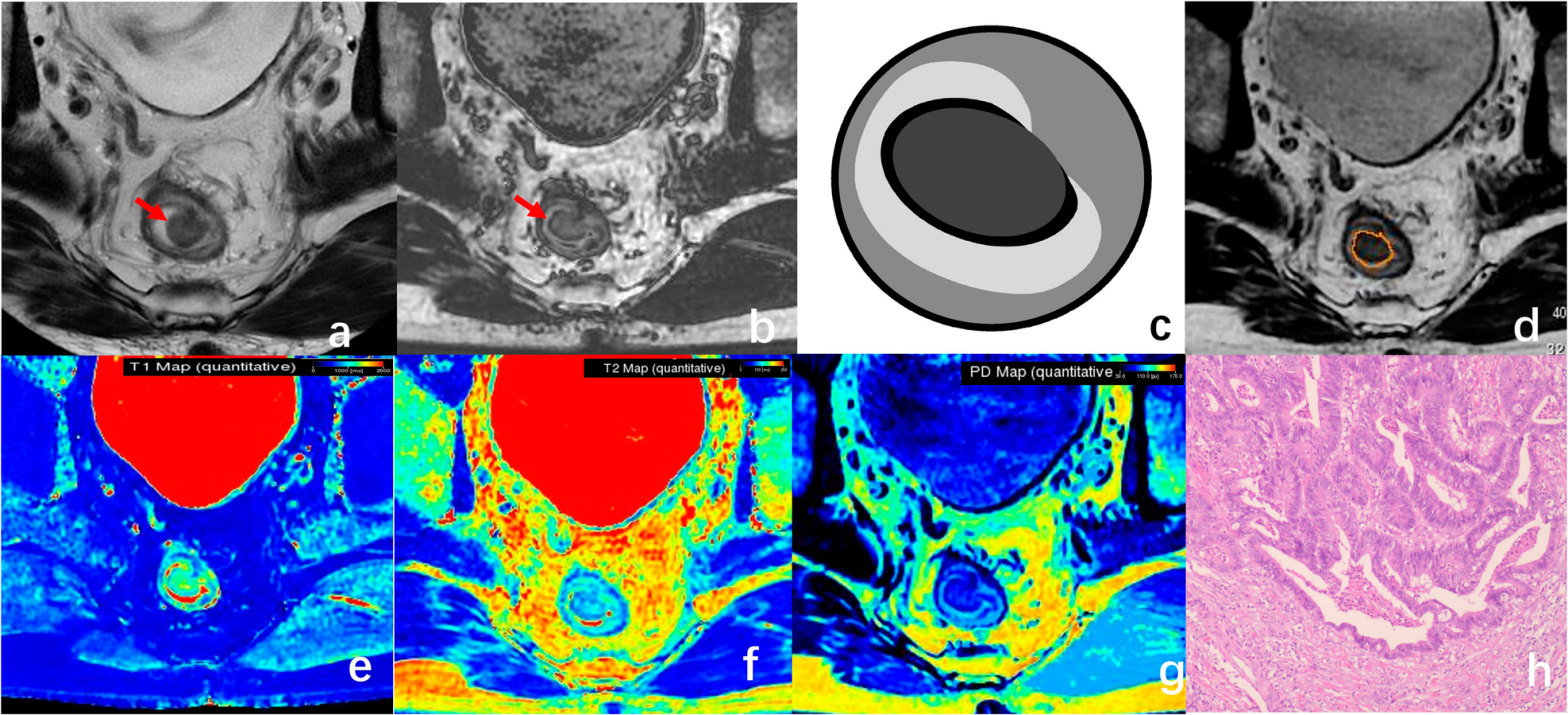
A 40-year-old man with T1 stage rectal tumor. The axial T2WI (**a**) and SyDIR images (**b**) reveal a villous tumor originating from the left rectal wall, characterized by a slender stalk and an intact muscularis propria. The schematic diagram of SyDIR (**c**) displays a black line at the interface between the tumor and the intestinal lumen. The tumor borders were manually delineated on the SyT2WI image (**d**). Quantitative magnetic resonance (MR) values (**e**–**g**) of the tumor were measured as follows: 1226 ms for T1, 103 ms for T2, and 76 pu for PD. Pathological analysis confirmed submucosal invasion (**h**, HE × 100)

For the T1 stage patient, the tumor grows through the muscularis mucosa and extends into the submucosa on conventional T2WI. The SyDIR image shows the dark band of the tumor (Fig. 2).

**Fig. 3:**
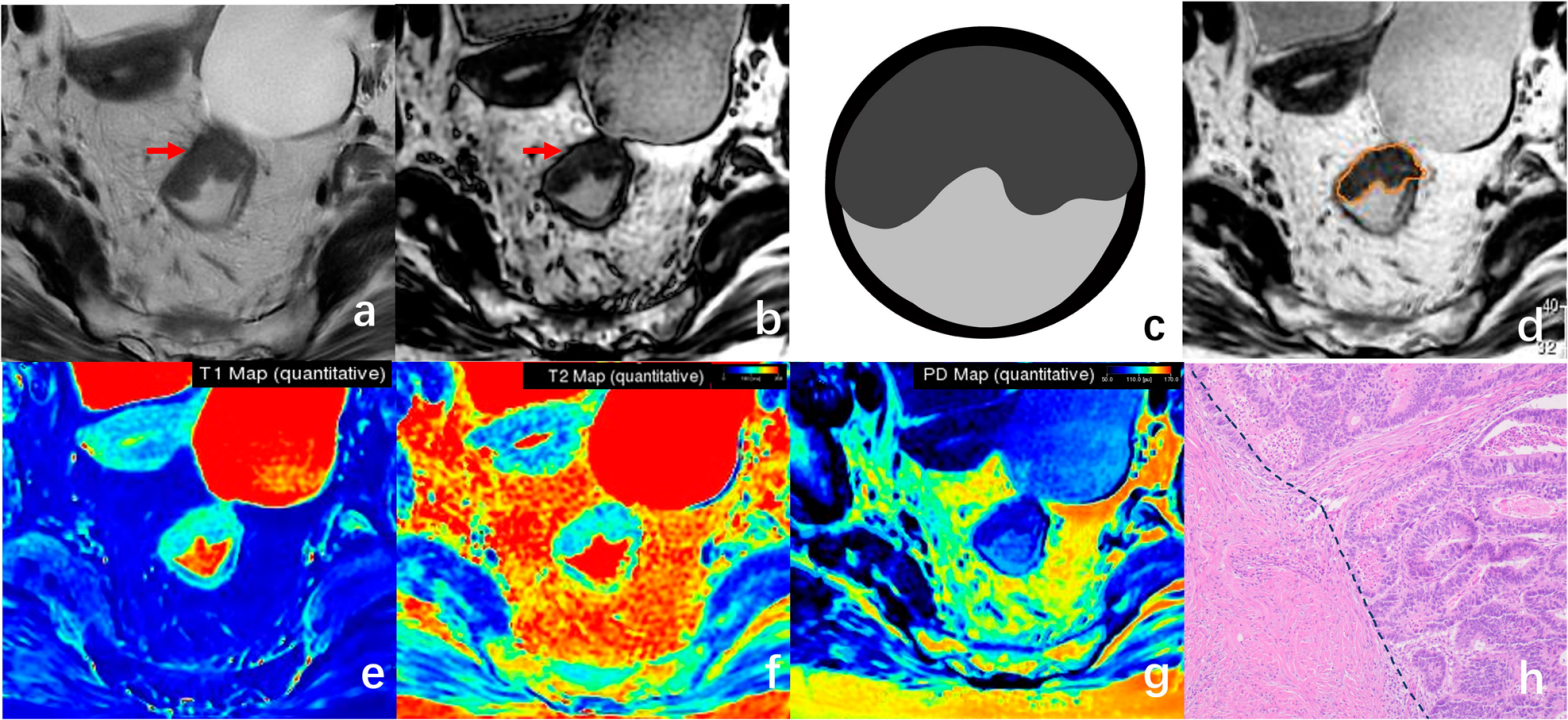
A 74-year-old woman with T2 stage rectal tumor. The axial T2WI image (**a**) reveals tumor infiltrating into the muscularis propria, characterized by focal blurring (arrow) and potential infiltrating into the mesorectal fat, which can lead to inaccurate staging. On the axial SyDIR image (**b**), the intact black ticking of the muscularis propria (arrow) is observed, indicating tumor involvement without infiltrating beyond the muscularis propria. The schematic diagram of SyDIR (**c**) displays a low signal boundary line between the tumor margin and the intrinsic muscular layer of the intestinal wall. The tumor border was manually delineated on the SyT2WI image (**d**). The quantitative magnetic resonance (MR) values (**e**–**g**) of the tumor were measured as follows: 1059 ms for T1, 113 ms for T2, and 78 pu for PD. Pathological examination confirmed the deep invasion of the muscular layer. The dashed line indicates the boundary between the adenocarcinoma tissue and the intrinsic muscular layer, with partial invasion of the muscular layer (**h**, HE × 100)

For the T2 stage patient, the tumor extends into the muscularis propria usually accompanied by spiculations in the mesorectum on T2WI. On the SyDIR image shows an intact muscularis propria, and the tumor didn’t extend into the mesorectum without forming nodular bands (Fig. 3).

**Fig. 4:**
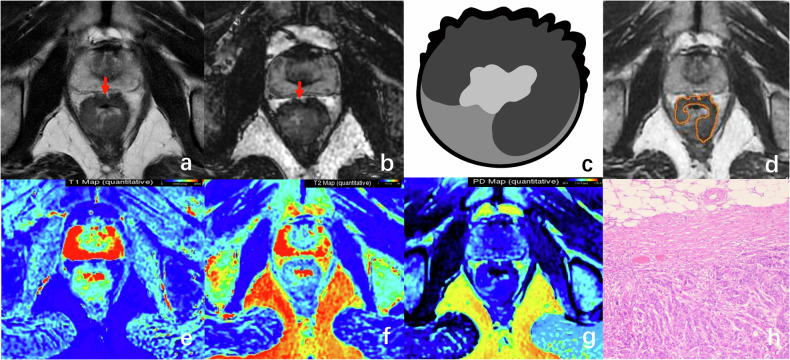
A 60-year-old man with T3 stage rectal tumor. The axial T2WI image (**a**) demonstrates tumor extension into the anterior mesorectal fat layer, characterized by blurred demarcation from the anterior prostate and discontinuity of the muscularis propria (arrow). On the axial SyDIR image (**b**), the presence of a nodular dark line indicates tumor infiltration through the muscularis propria into the mesorectal fat, while maintaining a distinct demarcation from the prostate in front of the rectal cancer lesion (arrow). The SyDIR schematic diagram (**c**) provides a clear visualization of the black line interface between the tumor margin and the mesorectal fat tissue, highlighting the boundary between them. The tumor border was manually outlined on the SyT2WI image (**d**). The quantitative magnetic resonance (MR) values (**e**–**g**) of the tumor were measured as follows: 1054 ms for T1, 81 ms for T2, and 75 pu for PD map. Pathological findings confirmed an extramural infiltration in this case (**h**, HE × 100)

For the T3 stage patient, the tumor infiltrates beyond the muscularis propria into the mesorectum on T2WI. SyDIR shows the tumor’s nodular dark line border infiltrating into the mesorectum (Fig. 4).

**Fig. 5:**
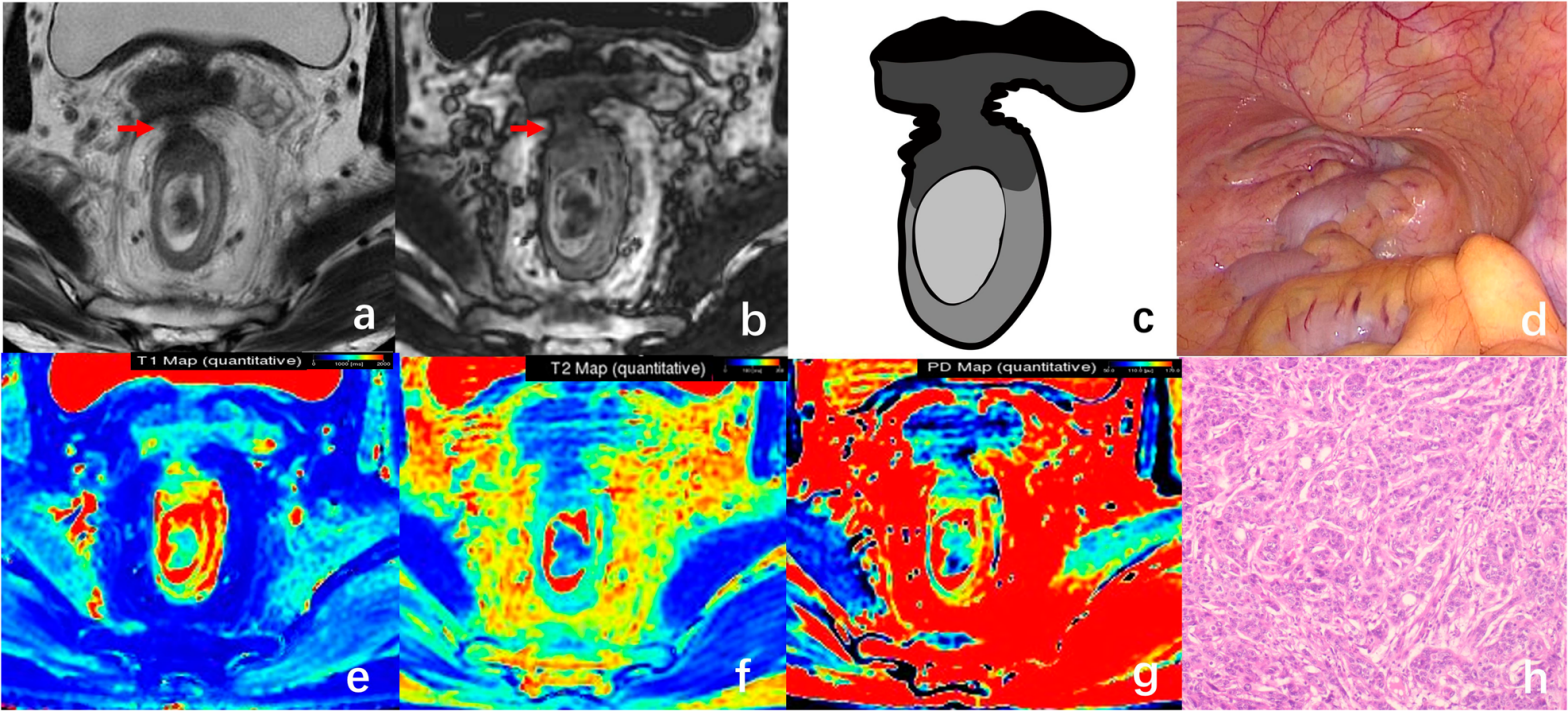
A 66-year-old man with T4 rectal tumor. The axial T2WI image (**a**) shows a tumor infiltrating beyond the muscularis propria, directly invading the seminal vesicle and possibly extending to the posterior wall of the bladder. On the axial SyDIR image (**b**), the tumor is clearly visible, encircling and invading the seminal vesicle gland, and extending into the bladder. The SyDIR schematic diagram (**c**) shows that the tumor, along with the seminal vesicle in front of the rectal cancer lesion, is encompassed by a black line. During laparoscopic total mesorectal excision (**d**), it was observed and confirmed that the tumor had invaded beyond the rectal fascia into the surrounding tissues. The quantitative MR values (**e**–**g**) of the tumor were measured as follows: 1137 ms for T1, 86 ms for T2, and 78 pu for PD. Pathological examination confirmed the presence of rectal adenocarcinoma (**h**, HE × 100)

For the T4 stage patient, the SyDIR clearly shows the dark line of tumor-invaded seminal vesicle glands and bladder (Fig. 5).

### Diagnostic efficacy of T stage and extramural infiltration in rectal cancer

The number of pathologically confirmed T1-2, T3, and T4 stage patients was 47, 38, and 27, respectively.

As shown in Table 3, compared to using T2WI alone, the consistency of T staging using a combination of T2WI and SyDIR was significantly improved from 0.547 to 0.780 for junior radiologist and from 0.738 to 0.834 for senior radiologists.

**Table 3:** The consistency between pathological and MRI staging results using T2WI alone and T2WI + SyDIR for the junior and senior radiologist

Readers	Method	MRI stage	Pathological stage			Sensitivity	Specificity	PPV	NPV	Kappa	Total Accuracy %
			T1-2	T3	T4						
Junior radiologist	T2WI	T1-2	31	3	2	88.6	92.5	86.1	81.6	0.547	70.5 (79/112)
		T3	12	32	6	74.4	73.9	64	82.3		
		T4	2	8	16	66.7	88.6	61.5	90.7		
	T2WI + SyDIR	T1-2	38	2	1	84.4	95.5	92.7	90.1	0.780	85.7 (96/112)
		T3	6	37	2	86	88.4	82.2	91.0		
		T4	1	4	21	87.5	94.3	80.8	96.5		
Senior radiologist	T2WI	T1-2	39	3	1	86.7	94.0	90.7	91.3	0.738	83.0 (93/112)
		T3	5	35	4	81.4	87.0	79.5	88.2		
		T4	1	5	19	79.2	93.2	76.0	94.3		
	T2WI + SyDIR	T1-2	40	2	0	88.9	97.0	95.2	92.9	0.834	89.3 (100/112)
		T3	4	39	3	90.7	89.9	84.8	93.9		
		T4	1	2	21	87.5	96.6	87.5	96.6		

*SyDIR* synthetic double inversion recovery, *T2WI* T2-weighted imaging, *PPV* positive predictive value, *NPV* negative predictive value

**Fig. 6:**
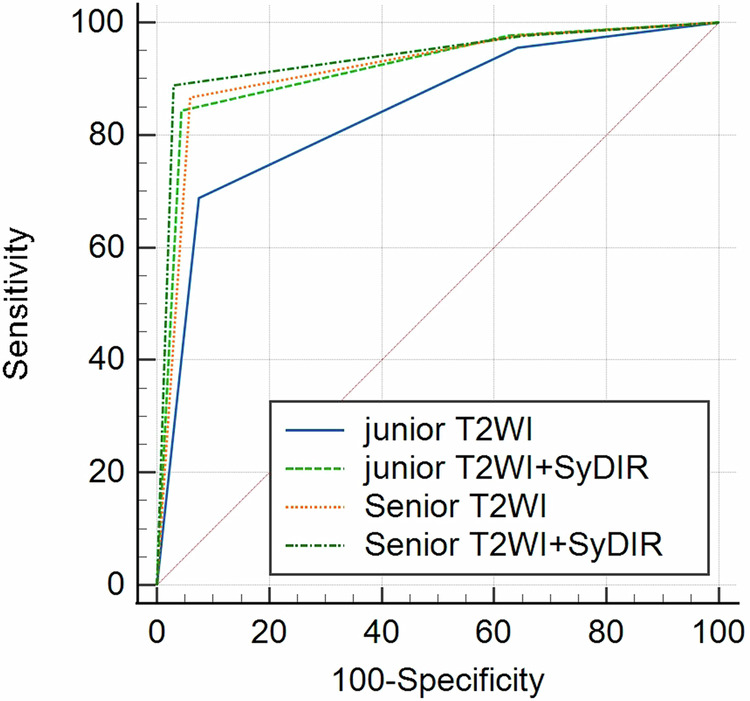
The ROC of extramural infiltration differentiation using T2WI alone and T2WI + SyDIR images for junior and senior radiologists

As shown in Fig. 6, the combination of T2WI and SyDIR also improved the diagnostic performance of extramural infiltration (T1-2 vs T3-4) for both junior and senior radiologists, AUC increased from 0.842 (95% confidence interval (CI): [0.716–0.904]) to 0.918 (95% CI: [0.851–0.962]) and from 0.917 (95% CI: [0.850–0.961]) to 0.938 (95% CI: [0.876–0.975]), respectively. AUCs for diagnostic performance of combined T2WI and SyDIR were superior to T2WI alone for junior radiologist (*p* = 0.0045, *z* = 2.839), while there is no difference with T2WI alone for senior radiologist (*p* = 0.9676, *z* = 0.0406).

### SyDIR quantitative parameters for evaluating the extramural infiltration

The differences in T1, T2, and PD values in (T1-2) and (T3-4) groups are shown in Table 4. The difference between the T2 values of the (T1-2) and (T3-4) groups was statistically significant (*p* < 0.001). T2 values were lower in the extramural infiltration group (T3-4) than in the non-infiltration group (T1-2). When the cutoff value was taken as 91 ms, the accuracy, sensitivity, and specificity of T2 values for diagnosing extramural infiltration were 74.1%, 64.4%, and 71.6%, respectively, with an AUC of 0.752 (95% CI, [0.661–0.828]).

**Table 4:** Differences of quantitative parameters between the (T1-2) and (T3-4) groups

Quantitative values of Synthetic MR	non-extramural infiltration group (T1-2) (*n* = 47)	extramural infiltration group (T3-4) (*n* = 65)	*p* value	ICC
T1 values (ms)				
reader 1	1313.87 ± 188.52	1314.27 ± 183.67	0.991	0.716
reader 2	1292.29 ± 166.24	1278.96 ± 168.29	0.681	
T2 values (ms)				
reader 1	94.16 ± 4.80	86.81 ± 4.62	< 0.001*	0.753
reader 2	93.09 ± 4.94	87.94 ± 5.47	< 0.001*	
PD values (pu)				
reader 1	67.16 ± 9.11	66.05 ± 7.62	0.486	0.810
reader 2	68.56 ± 8.0	66.48 ± 7.34	0.160	

*reader 1* Junior radiologist, *reader 2* Senior radiologist, *ICC* intraclass correlation coefficient, *PD* proton density, *pu* per unit

*p < 0.05

## Discussion

This study preliminarily explored the clinical value of synthetic DIR in evaluating the T staging of rectal cancer. In comparison to conventional T2WI, SyDIR provides comparable image quality. Furthermore, our study confirms that SyDIR adds value to T staging, with diagnostic accuracy increasing from 70.5% to 85.7% and 83% to 89.3% for junior and senior radiologists, respectively, when SyDIR is combined with T2WI. This combination also enhances the diagnostic performance of extramural infiltration, which is crucial for differentiating T1-2 from T3-4 stages, thus aiding in the selection of treatment options.

In primary rectal cancer, MR imaging is utilized to assist in staging, with HR-T2WI sequences playing a pivotal role. Although some studies have reported a high level of accuracy for T2WI in the staging of rectal cancer, these results have not been widely reproduced [6]. Conventional T2WI assessment of rectal cancer T-staging remains challenging due to the difficulty in distinguishing between T2 and early T3 stage tumors [7]. Penetration of small vessels into the muscle layers and fibrogenic reactions are common pitfalls that can cause a T2 tumor to be overstaged as a T3 tumor [7]. However, preoperative staging determines the choice of surgery and the use of radiotherapy or chemoradiotherapy, and preoperative differentiation of the T3 and T4 subgroups from the T1 and T2 subgroups is essential for treatment planning.

In previous studies, the DIR sequence employed two distinct inversion pulses to attenuate signals from different tissues within the imaging field [17]. However, this necessitated the prior determination of two suitable inversion recovery times through multiple scanning experiments to optimize tissue contrast, resulting in significant MR scanning time consumption [9, 12]. Unlike conventional approaches, SyDIR allows for the flexible adjustment of parameters such as TR, TE, and TI using post-processing algorithms, thereby enabling the selection of the most optimal image quality [18]. This innovative methodology expedites the examination process and mitigates the need for repeat scanning. In our study, the SyDIR sequence was specifically designed to enhance the contrast between tumor tissue, extraluminal fat, and intraluminal water. Initially, a prolonged TI was chosen to suppress the intraluminal water signal, followed by the meticulous optimization of an appropriate TI value to enhance the delineation of tumor boundaries and the interface with fat, leading to superior image quality. Importantly, our investigation involved an assessment of inter-reader agreement in evaluating SyDIR images, wherein we demonstrated the comparable performance of SyDIR to conventional T2WI based on metrics such as SNR, CNR, overall image quality, motion artifact presence, lesion conspicuity, and lesion edge sharpness.

In our study, we observed a characteristic “pencil-thin” rim appearance at the junction of the lesion and adjacent normal tissues, resulting from a well-known artifact of inversion recovery sequences known as the opposed-magnetization (or bounce point) artifact [19, 20]. This artifact has been utilized for diagnosing various conditions such as multiple sclerosis and heterotopic gray matter [12, 13, 21]. In this study, we find this phenomenon of SyDIR images is particularly helpful for radiologists, especially junior ones, in assessing T stage, given the significant improvement in diagnostic accuracy as well as AUC. This may be attributed to the enhanced soft tissue contrast provided by SyDIR. As we know, both T2 stage and T3 stage rectal cancers often exhibit fibrous bands resulting from inflammatory reactions in the surrounding tissues. These fibrous bands presented with similar signal intensity to that of the tumor on T2WI, which may lead radiologists to overstage T2 as T3, regardless of the experience of radiologists. With the application of SyDIR, the contrast between the tumor and fibrous tissues is enhanced, making it easier to identify tumor boundaries. This enhancement is visually represented as a distinct “black line” along the tumor’s edge on SyDIR.

Synthetic MR imaging can also provide quantified maps that display the absolute values of a patient’s physical properties [22]. Consistent with previous studies [23], our results also demonstrated that rectal cancer patients with higher T stages (T3-4) had a lower T2 value, with a cutoff of 91 ms, compared to patients with lower T stages (T1-2). We speculated that the increased cellularity, nuclear polymorphism, and nuclear/cytoplasmic ratio in highly aggressive rectal cancer may be responsible for the lower T2 value, which could be translated into a reduction in the extracellular fluid space [24]. Similar in other diseases [25, 26]. However, no significant difference in T1 and PD values was found in this study. The role of T1 and PD values in predicting rectal cancer T-staging needs to be further studied with larger sample sizes.

This study does have some limitations. First, it was a pilot study with a retrospective design and a small sample size. Selection bias might have occurred when some individuals were excluded because they had neoadjuvant radiation. A multicenter prospective study with bigger sample sizes is required for additional validation. Second, we only evaluated SyDIR’s image quality without comparing it with conventional DIR sequence images. In the future, we plan to apply the real DIR sequence to the rectum for further comparison and analysis. Thirdly, although SyDIR provides image quality comparable to conventional T2WI, the contrast between the tumor and the rectal wall is unsatisfactory when the tumor is large and located in the upper segment of the rectum. The SyDIR image quality was poor in six patients in this study. This may be attributed to the interference caused by respiratory motion artifacts and significant peritumoral edema observed in larger tumors.

## Conclusion

In conclusion, SyDIR can be utilized to characterize rectal cancer tumors. The combination of T2WI and SyDIR enhances the accuracy of extramural infiltration and improves T staging performance, which would be beneficial for selecting appropriate treatment options.

## Supplementary information

ELECTRONIC SUPPLEMENTARY MATERIAL

## Data Availability

The datasets generated during and/or analyzed during the current study are available from the corresponding author at reasonable request.

## Abbreviations

AUC: Area under the curve
CNR: Contrast-to-noise ratio
CI: Confidence interval
DIR: Double inversion recovery
HR-T2WI: High-resolution T2-weighted imaging
MRI: Magnetic resonance imaging
PD: Proton density
ROC: Receiver operating characteristic
ROI: Regions of interest
SNR: Signal-to-noise ratio
SyDIR: Synthetic double inversion recovery
SyMRI: Synthetic MRI
TI: Inversion time
T2WI: T2-weighted imaging
