# Supplementary material for: Synthetic double inversion recovery imaging for rectal cancer T staging evaluation: imaging quality and added value to T2-weighted imaging
Source: Insights Imaging. 2024 Oct 24;15:256. doi: 10.1186/s13244-024-01796-4 (PMC11502625; doi:10.1186/s13244-024-01796-4)
Supplement: Supplementary file 1 — ELECTRONIC SUPPLEMENTARY MATERIAL [file 13244_2024_1796_MOESM1_ESM.pdf]

Synthetic double inversion recovery imaging for rectal cancer T staging evaluation：imaging quality and added value to T2-weighted imaging

ELECTRONIC SUPPLEMENTARY MATERIAL

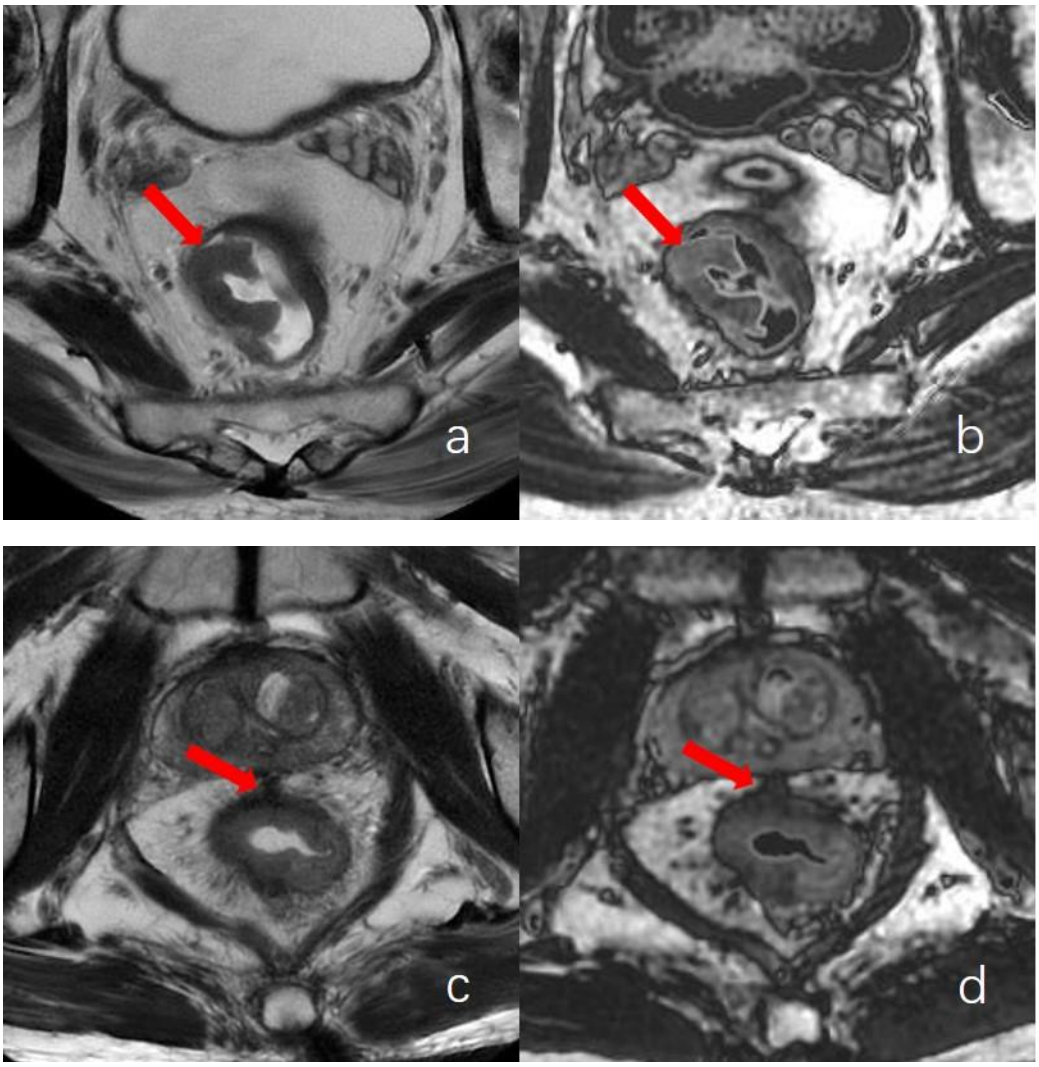

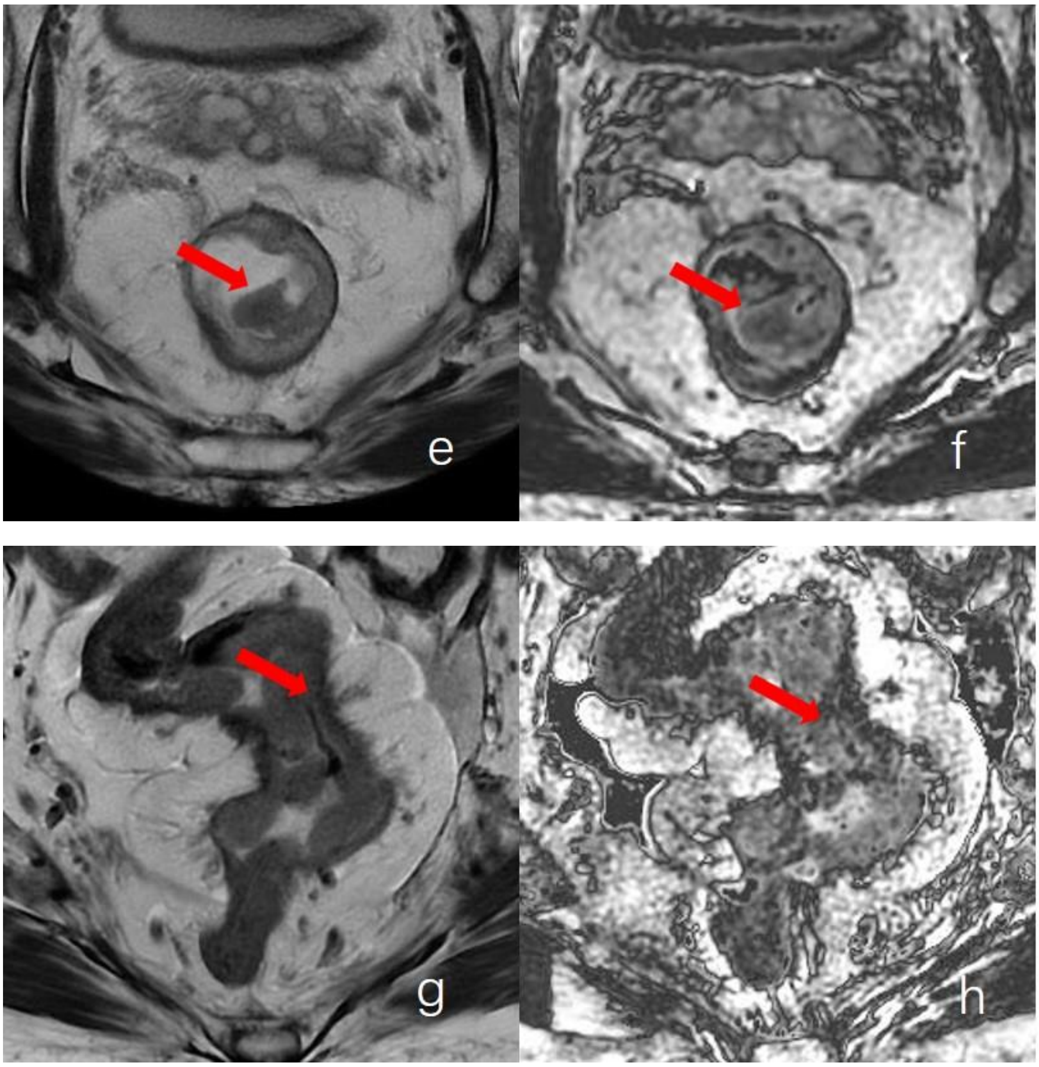

Supplementary Figure1: Schematic diagram of overall image quality rating.

Image b demonstrates a remarkably pronounced lesion conspicuity (lesion conspicuity=5), excellent lesion edge sharpness (lesion edge sharpness=5), absence of motion artifacts (presence of motion artifacts=5), and an overall image quality rating of 5 points.

Image d shows a relatively pronounced lesion conspicuity (lesion conspicuity=4), excellent lesion edge sharpness outside the lesion, and good lesion edge sharpness within the lesion (lesion edge sharpness=4). There are no motion artifacts (presence of motion artifacts=5), resulting in an overall image quality rating of 4 points.

Image f shows a moderate lesion appearance, requiring a comparison with Image e (T2WI) for lesion identification (lesion conspicuity=3). The lesion edge sharpness within the lesion is average, while the sharpness outside the lesion is relatively good (lesion edge sharpness=3). There are no motion artifacts (presence of motion artifacts=5). Consequently, the overall image quality rating is 3 points.

Image h shows a barely discernible lesion (lesion conspicuity=2), with average lesion edge sharpness both within and outside the lesion (lesion edge sharpness=2). There is mild presence of motion artifacts (presence of motion artifacts=4). Consequently, the overall image quality rating is 2 points.
